# Primary percutaneous stenting above the ampulla versus endoscopic drainage for unresectable malignant hilar biliary obstruction (TESLA RCT): study protocol for a multicenter randomized controlled trial

**DOI:** 10.1186/s12885-025-14158-0

**Published:** 2025-05-09

**Authors:** M. Rousian, V. van Verschuer, S. Franssen, D. Bijdevaate, R. P.H. Bokkers, A. E. Braat, J. de Bruijne, M. J. Bruno, M. C. Burgmans, O. M. van Delden, M. Dewulf, J. I. Erdmann, J. Hagendoorn, B. van der Holt, F. J.H. Hoogwater, A. Inderson, C. van der Leij, B. Mohseny, J. W. Poley, M. L.J. Smits, F. G.I. van Vilsteren, R. P. Voermans, I. A.J. Zijlstra, L. M.J.W. van Driel, B. Groot Koerkamp

**Affiliations:** 1 Department of Surgery, Erasmus MC Cancer Institute, Rotterdam, The Netherlands; 2 Department of Radiology, UMC Utrecht, University Medical Center, Utrecht, The Netherlands; 3 Department of Interventional Radiology, Erasmus MC, University Medical Center, Rotterdam, The Netherlands; 4 Department of Interventional Radiology, UMC Groningen, University Medical Center, Groningen, The Netherlands; 5 Department of Surgery, Leiden UMC, University Medical Center, Leiden, The Netherlands; 6 Department of Gastroenterology and Hepatology, UMC Utrecht, University Medical Center, Utrecht, The Netherlands; 7 Department of Gastroenterology and Hepatology, Erasmus MC, University Medical Center, Rotterdam, The Netherlands; 8 Department of Interventional Radiology, Leiden UMC, University Medical Center, Leiden, The Netherlands; 9 Department of Gastroenterology and Hepatology, Amsterdam University Medical center, University of Amsterdam, Amsterdam, The Netherlands; 10 Department of Surgery, Maastricht UMC, University Medical Center, Maastricht, The Netherlands; 11 Department of Surgery, Amsterdam University Medical center, University of Amsterdam, Amsterdam, The Netherlands; 12 Department of Surgery, UMC Utrecht, University Medical Center, Utrecht, The Netherlands; 13 Department of Hematology, Erasmus MC, University Medical Center, Rotterdam, The Netherlands; 14 Department of Surgery, UMC Groningen, University Medical Center, Groningen, The Netherlands; 15 Department of Gastroenterology and Hepatology, Leiden UMC, University Medical Center, Leiden, The Netherlands; 16 Department of Interventional Radiology, Maastricht UMC, University Medical Center, Maastricht, The Netherlands; 17 Department of Gastroenterology and Hepatology, Maastricht UMC, University Medical Center, Maastricht, The Netherlands; 18 Department of Interventional Radiology, UMC Utrecht, University Medical Center, Utrecht, The Netherlands; 19 Department of Gastroenterology and Hepatology, UMC Groningen, University Medical Center, Groningen, The Netherlands; 20 Department of Interventional Radiology, Amsterdam University Medical center, University of Amsterdam, Amsterdam, The Netherlands

**Keywords:** Malignant hilar biliary obstruction, Primary percutaneous stenting, Endoscopic biliary drainage, Randomized controlled trial, Major complications, Overall survival

## Abstract

**Background:**

Patients with malignant hilar biliary obstruction typically present with painless jaundice. They commonly have perihilar cholangiocarcinoma (pCCA), but also intrahepatic cholangiocarcinoma, gallbladder cancer, and metastases to the liver hilum can present with hilar biliary obstruction. Endoscopic biliary drainage is the standard of care in most centers. Many patients develop drainage-related complications after endoscopic biliary drainage for malignant hilar biliary obstruction, in particular cholangitis, resulting in reinterventions, clinical deterioration and a high mortality rate. Primary percutaneous stenting (PPS) aims to avoid bacterial contamination and reduce drainage-related complications. The aim of this randomized controlled trial is to compare PPS with endoscopic biliary drainage in patients with unresectable malignant hilar biliary obstruction.

**Methods:**

This multicenter phase 3 randomized controlled trial (TESLA RCT) will recruit 148 patients with unresectable malignant hilar biliary obstruction in six Dutch tertiary academic referral centers. Diagnosis of malignant hilar biliary obstruction is pathologically confirmed or determined as very likely by the multidisciplinary team. In the intervention arm, patients undergo primary percutaneous stenting with uncovered self-expandable metal stents without crossing the ampulla and without leaving an external drain. In the control arm patients undergo endoscopic biliary drainage according to international guidelines. The primary endpoint is major complications within 90 days after randomization. Secondary outcomes include technical success, reintervention rates, decrease of bilirubin levels, eligibility for palliative systemic treatment, quality of life, and overall survival.

**Discussion:**

The multicenter TESLA RCT compares primary percutaneous stenting with endoscopic biliary drainage in patients with unresectable malignant hilar biliary obstruction. First patient was randomized on August 9, 2023.

**Trial registration:**

Netherlands Trial Register (NL-OMON53463), registered on May 12, 2023, and Clinicaltrials.gov (NCT06671418), registered on November 1, 2024.

## Background

**Fig. 1:**
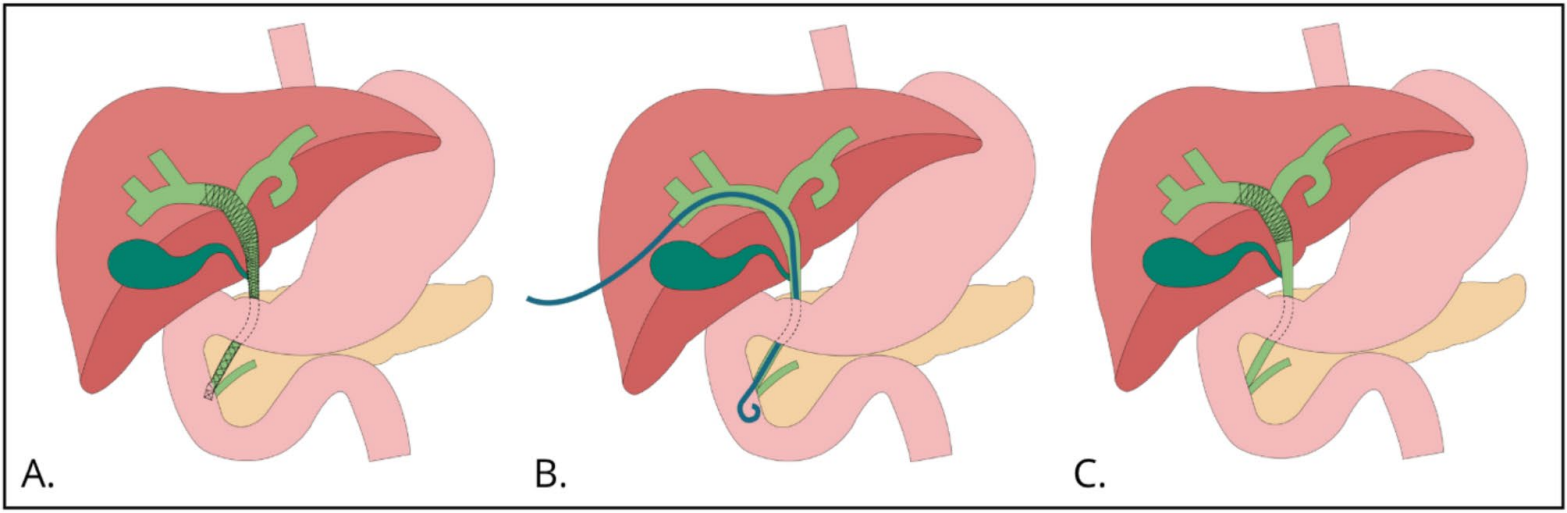
(**A**) Endoscopic biliary drainage (ERCP); (**B**) Percutaneous biliary drain (PBD); (**C**) Primary percutaneous stenting (PPS)

Malignant hilar biliary obstruction (MHBO) is most commonly caused by perihilar cholangiocarcinoma (pCCA), but can also be caused by intrahepatic cholangiocarcinoma, gallbladder cancer, and metastases from other malignancies to the liver hilum [1, 2]. Most patients with MHBO are ineligible for curative surgery due to the presence of distant metastases, locally advanced disease or poor performance status. In the palliative setting, the median overall survival of pCCA is approximately 5 months [3–5]. Painless jaundice is the typical presentation in patients with unresectable MHBO. Adequate biliary drainage is a prerequisite for effective palliative care including quality of life improvement and palliative systemic treatment [6]. The standard of care for palliative biliary drainage is through endoscopic retrograde cholangiopancreatography (ERCP) with plastic or self-expandable metal stents (SEMS; Fig. 1a). When ERCP has failed or is not feasible due to anatomical challenges (e.g., after a gastric bypass), percutaneous biliary drainage (PBD) is the preferred intervention (Fig. 1b) [7]. 

The main drawback of both ERCP and PBD is the high incidence of drainage-related infections caused by bacterial contamination of the biliary tract. This contamination is probably caused by the transpapillary approach followed by stents often placed across the ampulla, or internal-external bile duct drains placed percutaneously. Inadvertent instrumentation or contrast injection into undrained liver segments increase the risk of segmental cholangitis requiring reinterventions and often results in clinical deterioration. Consequently, most patients with unresectable MHBO never get systemic treatment and survival remains poor [4, 8, 9]. In a Dutch nationwide study of 186 patients with perihilar cholangiocarcinoma, 161 patients underwent ERCP and 24% of these patients required unplanned reintervention. The 90-day mortality of the entire cohort (ERCP and PBD) is 36%. Notably, no difference in 90-day mortality was found between initial ERCP and PBD [10]. In another cohort of 16.822 patients from the UK patients who underwent palliative PBD for biliary tract cancer had a median overall survival of 92 days after drainage [11]. 

We hypothesize that the poor outcomes of PBD and ERCP shown in prior studies are mainly due to bacterial contamination of the bile ducts caused by disruption of the ampulla. Primary percutaneous stenting (PPS) aims to maintain the sterility of the bile ducts as it avoids crossing the ampulla. The stents only cross the biliary duct obstruction with the distal end of the stent placed in the extrahepatic bile duct. By glueing the puncture trajectory after PPS the need for an external drain is omitted (Fig. 1c). This technique was investigated in our single center TESLA pilot study (MEC-2019-0789) of 67 patients with unresectable MHBO [12]. Cholangitis or pancreatitis were never observed after the initial PPS in all 67 patients. Other drainage-related severe complications were observed in only 17.9% and all were resolved with reinterventions; two patients (3.0%) developed acute cholecystitis, one patient (1.5%) a biliary leak, three patients (4.4%) hemorrhage, and six patients (9.0%) had persistent jaundice. Most patients (61.2%) started with palliative systemic treatment within four weeks after drainage. Palliative systemic treatment was never withheld due to complications of drainage or inadequate drainage. The median overall survival was 10.1 months (95% CI 7.9–14.4) with a 6-month OS of 68.7% (95% CI 58.4%-80.7%).

To validate the results of the non-randomized pilot study, we designed a multicenter, randomized controlled trial, the TESLA RCT. This study has been registered at Netherlands Trial Register (NL-OMON53463) and ClinicalTrials.gov (NCT06671418). The primary aim of this study is to determine whether PPS reduces major complications compared to ERCP in patients with unresectable MHBO.

## Methods

### Trial design

The TESLA RCT is a multicenter, phase 3, randomized controlled trial. All six Dutch academic centers treating patients with primary liver tumors have agreed to participate. Patients with unresectable MHBO are randomized for PPS with uncovered self-expandable metal stents (ucSEMS) without crossing the ampulla and without leaving an external drain (intervention; arm 1) or ERCP according to the American Society for Gastrointestinal Endoscopy (ASGE) guidelines of 2021 (control; arm 2). Randomization is performed in a 1:1 ratio using type of tumor (pCCA versus other types of malignancies) and center of inclusion as stratification factors through the web-based software tool for clinical research CASTOR EDC (version 2023.1.0.0). All participating centers must perform five PPS procedures in the TESLA pilot before participating in the TESLA RCT to minimize the impact of a potential learning curve for the intervention arm.

### Trial eligibility

Patients with unresectable MHBO and hyperbilirubinemia > 50 µmol/L (> 2.8 mg/dL) are screened for inclusion. All patients are discussed in a multidisciplinary team in an academic center to confirm ineligibility for both liver resection and liver transplantation. Unresectability can be due to local tumor extension (i.e., locally advanced disease), distant metastases, insufficient future liver remnant (FLR) function due to liver parenchyma disease, and/or poor patient performance. Ineligibility for liver transplantation is based on international guidelines [13]. Written informed consent is obtained. Cytological or histological confirmation of malignancy is recommended. However, study inclusion is also allowed if the multidisciplinary team agrees that the risk of malignancy is very high based on symptoms, imaging, and laboratory values.

Exclusion criteria include a high suspicion of benign disease, for example because of fluctuation or spontaneous decrease in bilirubin levels before the initiation of any treatment or a high IgG4. Previous biliary drainage attempts and clinical signs of cholangitis are other exclusion criteria. Cholangitis is defined as the presence of both fever (i.e., body temperature ≥ 38.5 °C) and leukocytosis (i.e., ≥ 10 × 10^9^/L) without clinical or radiological evidence of acute cholecystitis [14]. Patients who underwent an attempted, but failed ERCP are eligible only when no papillotomy or cannulation was performed.

### Treatment

In both treatment arms, the objective is to drain > 50% of non atrophic liver (ASGE guideline 2021) [4, 8, 9, 15]. A low threshold for placing more than one stent is recommended. Drainage of atrophic liver segments and liver segments with portal vein occlusion is avoided. Both procedures are performed under deep sedation with propofol and anesthesiologic monitoring.

### Intervention arm: PPS

**Fig. 2:**
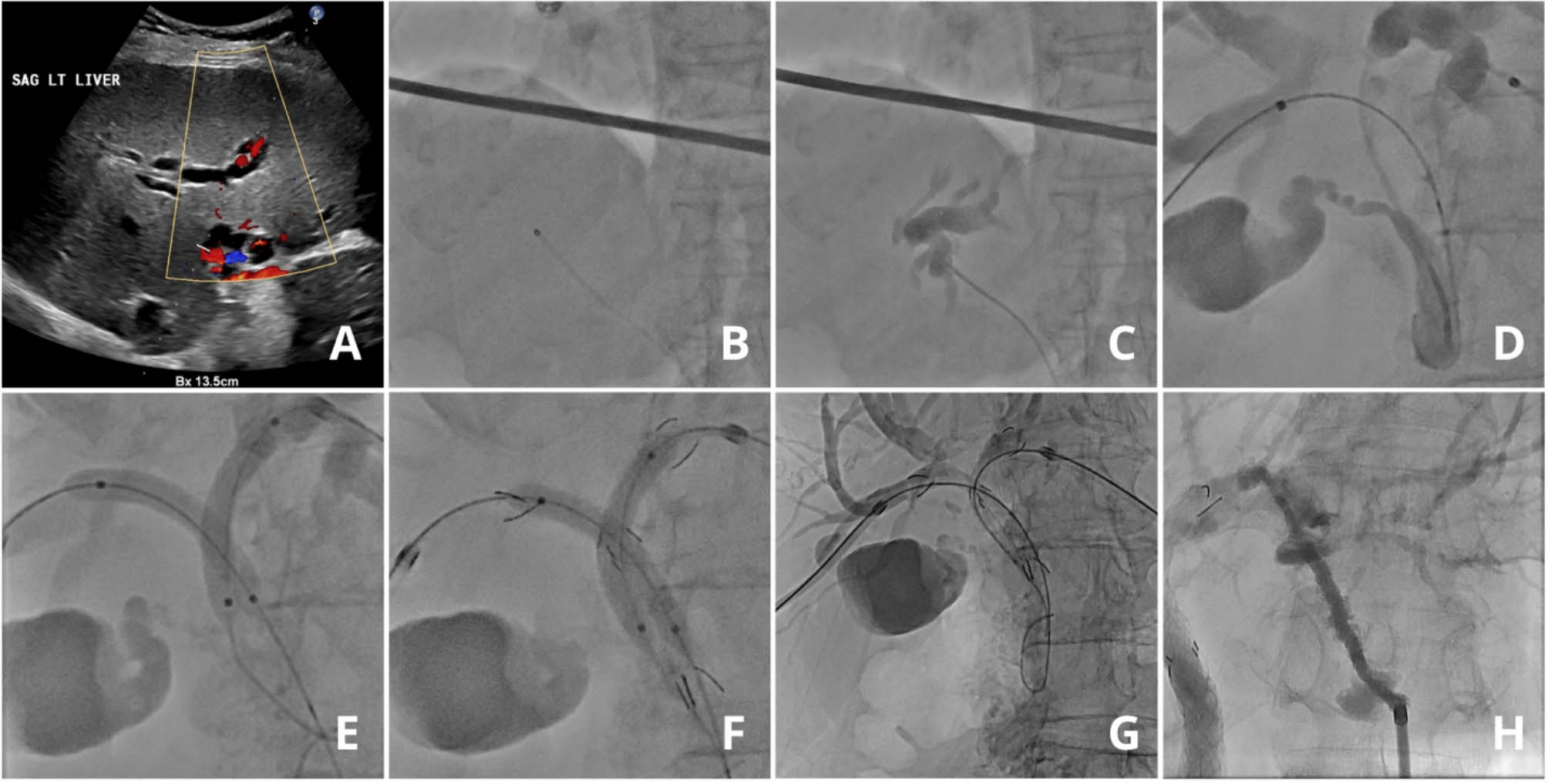
Step by step process of primary percutaneous stenting. Ultrasound-guided access to the biliary tree is obtained (**A**); and fluoroscopy confirms catheter placement in the bile duct (**B**); Contrast injection visualizes the biliary anatomy and obstruction (**C**); A guidewire is advanced, carefully passing the tumor while avoiding the ampulla (**D**); Balloon dilation is performed to widen the stricture (**E**); and stent insertion is performed, followed by post-dilation with two balloon catheters (**F**); Bilateral uncovered self-expanding metallic stents are deployed (**G**); and the puncture tract is sealed with Avitene (**H**)

After sterile exposure, percutaneous ultrasound- and fluoroscopy-guided access to the biliary tree is obtained (Fig. 2A-D). When pathological confirmation is lacking, percutaneous or endoluminal forceps biopsy or brush cytology is performed. Before stent insertion, the stricture is dilated with a balloon catheter of 6–8 mm that is pulled over the guidewire, as shown in Fig. 2E. Bilateral stenting involves kissing percutaneous transluminal angioplasty (PTA) with 6 mm. After balloon dilatation, an ucSEMS is placed ensuring adequate overlap with the unaffected bile ducts and without crossing the ampulla. After stent placement, post-dilation with a 6 to 8 mm kissing balloon catheter is conducted (Fig. 2F-G). The percutaneous transhepatic puncture tract is sealed with 0.5 gr Avitene™ (BD, NJ, USA) mixed with 8 ml of iodine contrast agent without leaving an external drain, as shown in Fig. 2H. Passing of the tumor may require several attempts and considerable time. Only when the tumor cannot be passed with a wire after repeated and prolonged attempts, an external biliary drain is placed. A second attempt of recanalization and stenting is performed after 3 to 5 days.

### Control arm: ERCP

ERCP is performed by a gastroenterologist who is experienced in performing ERCP in patients with MHBO. ERCP is performed according to the ASGE guidelines of 2021 [4, 8, 9, 15]. 

### Periprocedural care

#### Antibiotics

All patients receive a single intravenous dose of cefuroxime 1500 mg with metronidazole 500 mg during procedure. After the drainage procedure, all patients receive another five-day course of broad spectrum antibiotics (i.e., amoxicillin-clavulanic acid or ciprofloxacin). If bilirubin levels exceed 250 µmol/L (15 mg/dL) before procedure, its recommended to start prophylactic oral broad spectrum antibiotics at diagnosis to prevent spontaneous cholangitis prior to biliary drainage.

#### Discharge

The patient may be discharged once vital functions are both normal and stable, and pain is manageable (with or without oral analgesics). Patients in the intervention arm (i.e., PPS) are recommended to stay overnight due to the novel procedure, whereas patients in the endoscopic arm undergo ERCP as a day-case procedure, which is standard protocol in all centers in the Netherlands. Serum liver functions and hemoglobin are routinely checked before discharge; however, normalization of bilirubin is not required prior to discharge. The timing of discharge is determined at the discretion of the treating physician.

### Primary and secondary endpoints

The primary endpoint of this study is the proportion of patients with major complications within 90 days after randomization. Major complications are defined as any complication leading to prolonged hospital stay of more than 72 h; readmission to a hospital; any invasive reintervention, and/or death, within 90 days after randomization. Major complications include drainage-related complications (Table 1) and complications that do not appear to be drainage-related.

Secondary outcomes include primary and secondary technical success of stent placement at the initial drainage procedure, the number of reinterventions, admission days after drainage, patient-reported quality of life, bile cultures at initial drainage, and primary clinically successful drainage. Other secondary outcomes were the proportion of patients that started with palliative systemic treatment, 90-day mortality, and OS. Primary technical success was defined as successful passage of the stricture and stent placement without leaving an external drain during the initial drainage procedure. Secondary technical success was defined as successful passage of the stricture and stent placement during a second attempt 4–7 days after an unsuccessful initial procedure. Primary clinically successful drainage was defined as a bilirubin 50 µmol/L (> 2.8 mg/dL) or a reduction in bilirubin level of at least 50% within 14 days after the first drainage procedure.

**Table 1:** Definitions of severe drainage-related complications

Complication	Criteria
Cholangitis	Elevation in temperature ≥ 38,5 °C and Leukocytes ≥ 10 *10^9^/L, thought to have a biliary cause and requiring invasive intervention, without concomitant evidence of acute cholecystitis.
Acute cholecystitis	Radiologic evidence of cholecystitis, elevation in temperature more than 38.5 °C and Leukocytes ≥ 10 *10^9^/L, requiring emergency cholecystectomy or percutaneous drainage.
Persistent jaundice	Persistent elevated bilirubin levels after primary technically and clinically successful drainage had initially been obtained, without signs of cholangitis or cholecystitis, requiring a reintervention.
Acute pancreatitis	Two or more of the following criteria be met for the diagnosis of acute pancreatitis: abdominal pain suggestive of pancreatitis, serum amylase or lipase level greater than three times the upper normal value, or characteristic imaging findings.
Biliary leak	Symptomatic intra-abdominal bile leakage due to bile duct perforation or leakage from the puncture tract documented by any radiographic technique requiring intervention.
Haemorrhage	Clinical evidence of bleeding requiring blood transfusion or reintervention.

#### Follow-up

About 14 days after biliary drainage, serum bilirubin is measure and primary clinically successful drainage is assessed. Patients are recommended to have a consultation with a medical oncologist to discuss palliative systemic treatment. Quality of life is measured before treatment and at two weeks, one month, and three months follow-up using the EORTC QLQ-C30 and BIL21 questionnaires [16, 17]. The EORTC QLQ-C30 assesses quality of life specifically for cancer patients. The BIL21 focuses on symptoms related to patients with biliary tract cancer.

### Safety and monitoring

Adverse events (AEs) are defined as any undesirable event occurring to a subject during the study, regardless of whether they are related to the investigational intervention. AEs of grade III or higher according to the Clavien-Dindo classification, reported either spontaneously by the subject or observed by the investigator or study staff, will be systematically recorded. Serious Adverse Events (SAEs) are defined as any untoward medical occurrence or effect that results in death, is life threatening (at the time of the event), requires hospitalization or prolongation of existing inpatients’ hospitalization or results in persistent or significant disability or incapacity. SAEs will be reported via a web portal (Onderzoeksportaal) to the accredited Medical Ethics Review Committee (METC) within seven days for SAEs resulting in death or life-threatening events, with an additional eight days to complete the preliminary report. All other SAEs will be reported within 15 days of the sponsor’s first knowledge of the event. Reporting will continue for the duration of the study. All AEs will be followed until resolution or stabilization. An independent Data Safety Monitoring Board (DSMB) will oversee the safety data throughout the study. One non-binding interim analysis for superiority of the primary endpoint will be performed after the data of the first 60% randomized patients are available. The results by arm of the interim analysis will be presented to the DSMB, while the principal investigator will only receive results of both arms together.

### Data collection and statistical analysis

The handling of personal data is in compliance with the EU General Data Protection Regulation (GDPR) and the Dutch Act on Implementation of the General Data Protection Regulation (UAVG). Each patient is assigned a unique patient study number at enrolment, which is used to code the patient’s identity in the study documents. The investigator will keep a subject enrolment and identification log that contains the key to the code, i.e. a record of the personal identification data linked to each patient study number. This record is filed at the investigational site and should only be accessed by the investigator and the supporting site staff, and by representatives of the Sponsor or a regulatory agency for the purpose of monitoring visits or audits and inspections. All data will be entered in electronic case report forms using the electronic online database CASTOR EDC by trained data managers.

#### Sample size calculation

The sample size calculation was conducted for the primary outcome. The major complication rate in the control arm (ERCP) was estimated at 50% based on the literature. To detect a 50% reduction in the complication rate (to 25%) with 90% power and a 5% two-sided significance level, and with one non-binding interim analysis for superiority, a total of 148 patients will need to be randomized. The sample size calculation was performed using East software (version 6.5, Cytel Inc., Cambridge, MA). The estimated accrual period is three years. Total study duration is 3.5 years with 6 months follow-up after inclusion of the last patient.

#### Statistical analysis

The primary analysis will be conducted on an intention-to-treat basis, i.e. patients will be analyzed according to the arm they were assigned to at randomization. The proportion of patients with a major complication within 90 days after randomization and 95% confidence interval will be calculated by randomization arm. The difference in proportions of patients with major complications at the interim and final analysis will be analyzed using East version 6.5, using the Interim Monitoring option. For other dichotomous outcomes, proportions will be compared between treatment arms using the Chi-squared test. For continuous outcomes, including absolute and relative decrease in bilirubin levels, the Mann-Whitney U test will be used to compare treatment arms. Repeated measurements, including quality of life scores over time, will be analyzed using linear mixed models to account for intra-patient correlation with adjustments for baseline values. Survival outcomes will be analyzed using the Kaplan-Meier method, with survival differences at 3 and 6 months after randomization between groups assessed using Fisher’s exact test (when no loss to follow up occurs), or the logrank test, whichever applicable. A p-value of < 0.05 will be considered statistically significant. All statistical analyses will be performed using validated software, including East version 6.5 for interim monitoring.

## Discussion

The TESLA RCT is a multicenter phase 3 RCT comparing PPS with ERCP in patients with unresectable MHBO. Guidelines are inconsistent about the optimal approach for palliative drainage of MHBO. Clinical guidelines of the ASGE 2021 and ACG 2023 suggest either ERCP or PBD in patients with unresectable MHBO undergoing palliative drainage [15, 18]. The final decision should be based on patient preferences, disease characteristics, and local expertise. The NCCN guidelines for biliary cancer do not express a preference for ERCP or PBD [19]. Typically, PBD involves internal-external drainage with a plastic drain. PPS as conducted in the present study involves a distinct approach with stent placement without crossing the ampulla and without leaving an external biliary drain, in a single intervention.

Several studies have investigated ERCP and PBD (with internal-external drain) for both unresectable and resectable MHBO. However, patient outcomes have been consistently poor with both approaches. Up to date, only two randomized controlled trials have investigated biliary drainage in patients with resectable pCCA. Both trials were discontinued prematurely. The DRAINAGE trial randomized 54 patients with resectable pCCA from four academic Dutch centers. This study demonstrated a 90-day mortality of 14% and 40% after ERCP and PBD, respectively. Although not statistically significant (*p* = 0.08), the mortality difference was considered clinically relevant, and the study was prematurely stopped. The INTERCPT trial from the United States evaluated patients with MHBO across all disease stages. This trial included only 13 patients who underwent ERCP (*N* = 8) or PBD (*N* = 5) and was discontinued due to slow accrual. Mortality after drainage in the INTERCPT trial was high with a 3-month mortality rate of 50% after ERCP, and 80% after PBD [20, 21]. 

In addition, several retrospective studies have explored percutaneous SEMS placement in patients with MHBO. Paik et al. showed an improvement of technical success rates for PDB compared to ERCP in patients with Bismuth III or IV pCCA (92.7% vs. 77.3%, *p* = 0.049) [22]. The risk of drainage-related complications was comparable between both ERCP and PBD (29.5% vs. 31.7%, *p* = 0.83). Similarly, Thornton et al. described outcomes of primary percutaneous SEMS placement in 71 patients receiving PPS at Memorial Sloan Kettering Cancer Center (MSKCC) [23]. Major drainage related complications occurred in 14% (*N* = 10). It is important to note that in both studies, an external biliary drain was left in place, and stent placement (plastic or metal) either crossed the ampulla or ampulla involvement was not reported.

Notably, both studies utilized external biliary drainage with SEMS placed across the ampulla, whereas plastic stent (PS) placement in prior reports either avoided ampullary involvement or did not specify this anatomical relationship. This distinction may be clinically relevant, as SEMS placement across the ampulla– analogous to our earlier findings in resectable perihilar cholangiocarcinoma [4]– could enhance drainage efficacy through improved stent patency compared to PS.

PPS as an alternative to ERCP and PBD aims to prevent bacterial contamination of the biliary tract. This approach was investigated in the TESLA pilot study (MEC-2019-0789) involving 67 patients with MHBO and without prior drainage attempts (i.e. uncolonized biliary tract). Most patients were diagnosed with perihilar cholangiocarcinoma (40.3%), followed by intrahepatic cholangiocarcinoma (34.3%), gallbladder cancer (13.4%), liver metastases (10.5%), and hepatocellular carcinoma (1.5%). PPS showed a technical success rate of 98.5% at the initial procedure. Severe drainage-related complications occurred in 17.9% of patients. There were no cases of cholangitis or pancreatitis observed. Complications included acute cholecystitis (3.0%), biliary leak (1.5%), hemorrhage (4.4%), and persistent jaundice requiring additional stenting (9.0%). No drainage-related mortality was reported. Palliative systemic treatment was initiated in 62.7% of patients, and the median overall survival was 10.1 months (95% CI 7.9–14.4) [12]. (TESLA pilot study, submitted) The single-center and single-arm setting may limit the reproducibility of the TESLA pilot study. The participating interventional radiologists have specific expertise on treatment of hilar obstructions which may have had a favorable impact on the outcome. Comparisons with historical ERCP and PBD data may underestimate the quality and outcomes of the current procedures. Especially ERCP outcomes may have improved over time due to novel approaches such as suprapapillary stenting and centralization. Prior research has shown that SEMS placement is associated with lower stent failure rates compared to plastic stents in patients with resectable perihilar cholangiocarcinoma, potentially contributing to drainage outcomes [24]. 

This study has several limitations. The most important challenge in this study remains the referral of patients before they have undergone biliary drainage attempts. Participating centers will need to convince their potential referring centers to refer patients with suspected MHBO timely and undrained. After referral, multidisciplinary assessment of eligibility for the study needs to be facilitated. Reproducibility of successful trial results may be challenging, as the success of biliary interventions partly depends on the expertise of the interventional radiologist and the interventional gastroenterologist. As PPS is a novel intervention, all participating centers are required to perform five procedures following the TESLA pilot protocol before contributing to the TESLA RCT, ensuring procedural consistency and reducing the impact of operator variability on trial outcomes. Another potential limitation is that the protocol allows for placement of ucSEMS without pathological confirmation. To reduce the risk of stent placement in patients with benign disease, well-defined inclusion and exclusion criteria are implemented, including case discussion in a multidisciplinary team at an academic center, and monitoring of bilirubin trends before the procedure.

## Conclusion

In conclusion, the TESLA RCT is designed to investigate whether PPS is superior to ERCP in patients with unresectable MHBO. By addressing the limitations of traditional ERCP and PBD, particularly the risk of bacterial contamination associated with drainage-related complications, PPS without crossing the ampulla and without leaving an internal-external drain has the potential to improve patient outcomes. The multicenter randomized design of the TESLA RCT, with standardized procedural expertise, aims to provide robust evidence that could change the standard of care for patients with malignant hilar biliary obstruction.

## Data Availability

No datasets were generated or analysed during the current study.

## Declarations

### Trial status

The TESLA RCT opened for accrual on May 12, 2023, and the first patient was randomized on August 9, 2023. As of April 18, 2025, 4 participating Dutch centers are open for inclusion and 67 patients have been randomized.

## Abbreviations

AE: Adverse Event
ASGE: American Society for Gastrointestinal Endoscopy
CI: Confidence Interval
CT: Computed Tomography
DSMB: Data Safety Monitoring Board
ERCP: Endoscopic Retrograde Cholangiopancreatography
EORTC QLQ: European Organization for Research and Treatment of Cancer Quality of Life Questionnaire
GDPR: General Data Protection Regulation
MHBO: Malignant Hilar Biliary Obstruction
METC: Medical Ethics Review Committee
MSKCC: Memorial Sloan Kettering Cancer Center
NCCN: National Comprehensive Cancer Network
OS: Overall Survival
PBD: Percutaneous Biliary Drainage
PCCA: Perihilar Cholangiocarcinoma
PPS: Primary Percutaneous Stenting
RCT: Randomized Controlled Trial
SAE: Serious Adverse Event
SEMS: Self-Expandable Metal Stent
UAVG: Dutch Act on Implementation of the General Data Protection Regulation
UCSEMS: Uncovered Self-Expandable Metal Stent

## References

[1] Alvaro D, Bragazzi MC, Benedetti A, Fabris L, Fava G, Invernizzi P, et al. Cholangiocarcinoma in Italy: A National survey on clinical characteristics, diagnostic modalities and treatment. Results from the cholangiocarcinoma committee of the Italian association for the study of liver disease. Dig Liver Dis. 2011;43(1):60–5. 20580332 10.1016/j.dld.2010.05.002

[2] Hawkins WG, DeMatteo RP, Jarnagin WR, Ben-Porat L, Blumgart LH, Fong Y. Jaundice predicts advanced disease and early mortality in patients with gallbladder cancer. Ann Surg Oncol. 2004;11(3):310–5. 14993027 10.1245/aso.2004.03.011

[3] Chaiteerakij R, Harmsen WS, Marrero CR, Aboelsoud MM, Ndzengue A, Kaiya J, et al. A new clinically based staging system for Perihilar cholangiocarcinoma. Am J Gastroenterol. 2014;109(12):1881–90. 25384902 10.1038/ajg.2014.327 PMC4341961

[4] van Keulen AM, Franssen S, van der Geest LG, de Boer MT, Coenraad M, van Driel L, et al. Nationwide treatment and outcomes of Perihilar cholangiocarcinoma. Liver Int. 2021;41(8):1945–53. 33641214 10.1111/liv.14856 PMC8359996

[5] Jarnagin WR, Fong Y, DeMatteo RP, Gonen M, Burke EC, Bodniewicz BJ, et al. Staging, resectability, and outcome in 225 patients with hilar cholangiocarcinoma. Ann Surg. 2001;234(4):507–17. discussion 17– 9. 11573044 10.1097/00000658-200110000-00010 PMC1422074

[6] Blechacz B, Cholangiocarcinoma. Current knowledge and new developments. Gut Liver. 2017;11(1):13–26. 27928095 10.5009/gnl15568 PMC5221857

[7] Valle JW, Borbath I, Khan SA, Huguet F, Gruenberger T, Arnold D. On behalf of the EGC. Biliary cancer: ESMO clinical practice guidelines for diagnosis, treatment and follow-up. Ann Oncol. 2016;27:v28–37. 27664259 10.1093/annonc/mdw324

[8] Committee ASP, Jue TL, Storm AC, Naveed M, Fishman DS, Qumseya BJ, et al. ASGE guideline on the role of endoscopy in the management of benign and malignant gastroduodenal obstruction. Gastrointest Endosc. 2021;93(2):309–22. e4. 33168194 10.1016/j.gie.2020.07.063

[9] Cillo U, Fondevila C, Donadon M, Gringeri E, Mocchegiani F, Schlitt HJ, et al. Surgery for cholangiocarcinoma. Liver Int. 2019;39(1):143–55. 30843343 10.1111/liv.14089 PMC6563077

[10] Keulen AV, Gaspersz MP, van Vugt JLA, Roos E, Olthof PB, Coelen RJS, et al. Success, complication, and mortality rates of initial biliary drainage in patients with unresectable Perihilar cholangiocarcinoma. Surgery. 2022;172(6):1606–13. 35989132 10.1016/j.surg.2022.06.028

[11] Rees J, Mytton J, Evison F, Mangat KS, Patel P, Trudgill N. The outcomes of biliary drainage by percutaneous transhepatic cholangiography for the palliation of malignant biliary obstruction in England between 2001 and 2014: a retrospective cohort study. Bmj Open. 2020;10(1):e033576. 31980509 10.1136/bmjopen-2019-033576 PMC7045186

[12] Franssen S, van Driel LMJW, Moelker A, Groot Koerkamp B. Primary percutaneous stenting above the ampulla for palliative biliary drainage of malignant hilar biliary obstruction. J Clin Oncol. 2023;41(4suppl):527.

[13] European Association for the Study of the Liver. Electronic address Eee. EASL clinical practice guidelines: liver transplantation. J Hepatol. 2016;64(2):433–85. 26597456 10.1016/j.jhep.2015.10.006

[14] Wiggers JK, Coelen RJ, Rauws EA, van Delden OM, van Eijck CH, de Jonge J, et al. Preoperative endoscopic versus percutaneous transhepatic biliary drainage in potentially resectable Perihilar cholangiocarcinoma (DRAINAGE trial): design and rationale of a randomized controlled trial. BMC Gastroenterol. 2015;15:20. 25887103 10.1186/s12876-015-0251-0 PMC4332425

[15] Qumseya BJ, Jamil LH, Elmunzer BJ, Riaz A, Ceppa EP, Thosani NC, et al. ASGE guideline on the role of endoscopy in the management of malignant hilar obstruction. Gastrointest Endosc. 2021;94(2):222–34. e22. 34023067 10.1016/j.gie.2020.12.035

[16] Aaronson NK, Ahmedzai S, Bergman B, Bullinger M, Cull A, Duez NJ, et al. The European organization for research and treatment of Cancer QLQ-C30: a quality-of-life instrument for use in international clinical trials in oncology. J Natl Cancer Inst. 1993;85(5):365–76. 8433390 10.1093/jnci/85.5.365

[17] Kaupp-Roberts SD, Yadegarfar G, Friend E, O’Donnell CM, Valle JW, Byrne C, et al. Validation of the EORTC QLQ-BIL21 questionnaire for measuring quality of life in patients with cholangiocarcinoma and cancer of the gallbladder. Br J Cancer. 2016;115(9):1032–8. 27673364 10.1038/bjc.2016.284 PMC5117782

[18] Elmunzer BJ, Maranki JL, Gomez V, Tavakkoli A, Sauer BG, Limketkai BN, et al. ACG clinical guideline: diagnosis and management of biliary strictures. Am J Gastroenterol. 2023;118(3):405–26. 36863037 10.14309/ajg.0000000000002190

[19] Benson AB, D’Angelica MI, Abbott DE, Anaya DA, Anders R, Are C, et al. Hepatobiliary cancers, version 2.2021, NCCN clinical practice guidelines in oncology. J Natl Compr Canc Netw. 2021;19(5):541–65. 34030131 10.6004/jnccn.2021.0022

[20] Coelen RJS, Roos E, Wiggers JK, Besselink MG, Buis CI, Busch ORC, et al. Endoscopic versus percutaneous biliary drainage in patients with resectable Perihilar cholangiocarcinoma: a multicentre, randomised controlled trial. Lancet Gastroenterol Hepatol. 2018;3(10):681–90. 30122355 10.1016/S2468-1253(18)30234-6

[21] Elmunzer BJ, Smith ZL, Tarnasky P, Wang AY, Yachimski P, Banovac F, et al. An unsuccessful randomized trial of percutaneous vs endoscopic drainage of suspected malignant hilar obstruction. Clin Gastroenterol Hepatol. 2021;19(6):1282–4. 32454259 10.1016/j.cgh.2020.05.035 PMC8776356

[22] Paik WH, Park YS, Hwang JH, Lee SH, Yoon CJ, Kang SG, et al. Palliative treatment with self-expandable metallic stents in patients with advanced type III or IV hilar cholangiocarcinoma: a percutaneous versus endoscopic approach. Gastrointest Endosc. 2009;69(1):55–62. 18657806 10.1016/j.gie.2008.04.005

[23] Thornton RH, Frank BS, Covey AM, Maybody M, Solomon SB, Getrajdman GI, Brown KT. Catheter-free survival after primary percutaneous stenting of malignant bile duct obstruction. AJR Am J Roentgenol. 2011;197(3):W514–8. 21862781 10.2214/AJR.10.6069

[24] de Jong DM, Gilbert TM, Nooijen LE, Braunwarth E, Ninkovic M, Primavesi F, et al. Preoperative endoscopic biliary drainage by metal versus plastic stents for resectable Perihilar cholangiocarcinoma. Gastrointest Endosc. 2024;99(4):566–76. e8. 37866710 10.1016/j.gie.2023.10.041

